# Draft genome sequence of *Rossellomorea marisflavi* DL-A, a malathion-degrading bacterium

**DOI:** 10.1128/mra.00220-25

**Published:** 2025-06-10

**Authors:** Kiana Pillay, Srimanti Duttagupta, Gayatri Basapuram, Avishek Dutta

**Affiliations:** 1 Department of Microbiology, University of Georgia 189270, Athens, Georgia, USA; 2 Department of Geology, University of Georgia 1355, Athens, Georgia, USA; 3 Savannah River Ecology Laboratory, University of Georgia 17219, Aiken, South Carolina, USA; California State University San Marcos, San Marcos, California, USA

**Keywords:** *Rossellomorea*, malathion-degrading bacterium, river water

## Abstract

This study presents the draft genome of *Rossellomorea marisflavi* DL-A, a malathion-degrading bacterium isolated from the North Oconee River in Athens, GA, USA. The DL-A genome is 4.27 Mb in size and comprises 4,271 coding sequences with proteins.

## ANNOUNCEMENT

**Fig 1:**
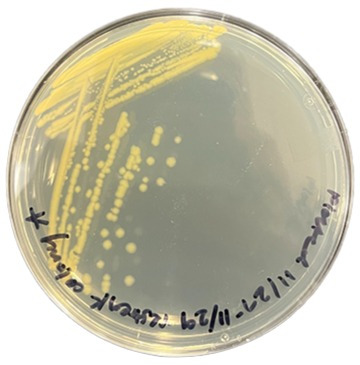
Yellow-pigmented colonies of strain DL-A grown on LB media.

Microbial diversity in riverine systems determines the fate and transport of organic contaminants (1). Studies suggest that the biotransformation of organic contaminants can help reduce insecticide pollution in the environment (2). The North Oconee River, located in northeastern Georgia, USA, flows through a diverse landscape that includes urban, industrial, and agricultural zones, making it a critical waterway for assessing contaminant degradation processes. Previous studies have detected traces of malathion, a widely used organophosphate insecticide, in the North Oconee River, highlighting the potential for natural microbial communities to facilitate its degradation (1). As a result, this study aimed to identify potential malathion-degrading bacterial strains in the river. One liter of water sample was collected in a sterilized polypropylene bottle from a depth of 1 m from the North Oconee River (33°57′24.0″N 83°22′01.2″W) on 9th October 2023 and transported to the lab and stored at 4℃. Within 24 hours, the water sample was plated on Luria-Bertani agar media and incubated for 24 hours at 30°C. Colonies were streaked for isolation and assessed for the ability to degrade malathion. Each isolated colony was grown in the presence of malathion, and the degradation of malathion was assessed using gas chromatography-mass spectrometry. The bacterial strain DL-A (Fig. 1) was found to be one of the most potent malathion-degrading strains (~41% degradation of 1% malathion in 24 hours at 30℃). In this study, whole genome sequencing was conducted for the DL-A strain, and we present the following results.

DL-A was identified as *Rossellomorea* sp. based on 16S rRNA gene sequencing (GenBank accession PV167751). For 16S rRNA gene sequencing, colonies were submitted to Azenta Life Sciences Inc. (South Plainfield, NJ, USA), where Sanger Sequencing was conducted. Briefly, the colonies underwent NaOH lysis, followed by amplification of the V1–V9 region of the 16S rRNA gene, enzymatic cleanup, and dye-terminator sequencing using Applied Biosystems BigDye version 3.1 on Applied Biosystems’ 3730xl DNA Analyzer. Sequencing-specific primers were used to generate bidirectional reads. For genome analysis, DNA was extracted using the DNeasy 96 PowerSoil Pro QIAcube HT Kit on a QIAcube HT platform as per the manufacturer’s protocol. Briefly, overnight cultures were grown and placed in a PowerBead Pro tube, then processed using the Tissuelyser II for cell lysis. The DNA from the resulting supernatant was purified using the QIAcube HT instrument. The extracted DNA was measured using the DeNovix dsDNA Broad Range assay kit in the DeNovix QFX fluorometer and sent to Novogene (USA) for sequencing. The sequencing library preparation was conducted using the Rapid Plus DNA Lib Prep Kit (ABclonal). Quantified libraries were sequenced on the Illumina NovaSeq 6000 platform with a 150 bp paired-end-read protocol. For genome assembly and other downstream analyses, the default parameters were used for all the software, unless otherwise mentioned. The raw genome sequence data were quality filtered using Fastp (version 0.23.2) (3), and the filtered reads were assembled with SPAdes (version 3.15.5) with --isolate option (4). The quality of the assembly was evaluated using QUAST (version 5.2.0) (5). CheckM (version 1.2.2) (6) was used to assess the genome’s completeness and contamination, and GTDB-Tk (version 2.3.2) (7) was used for taxonomic classification. The summary results of the sequencing, assembly, and annotation are presented in Table 1.

**TABLE 1:** Sequencing, *de novo* assembly, and annotation summary results of the sequenced genome

Parameters	*Rossellomorea marisflavi* DL-A
Number of raw reads	8,999,674 (forward + reverse reads)
Number of filtered reads	8,681,450 (forward + reverse reads)
Mean read length (bp) after filtering	149.9
*N*_50_	415,286
No. of contigs	78
Assembly size	4,265,157
Estimated sequencing depth	~305
Estimated completeness (%)	99.34
GC content (%)	48.23
No. of CDSs with protein^*a*^	4,271
No. of rRNAs^*a*^	3, 3, 2 (5S, 16S, 23S)
No. of tRNAs^*a*^	76
No. of ncRNAs^*a*^	5

^*a*^ These values are from the NCBI Prokaryotic Genome Annotation Pipeline 6.9 (8), where contigs of size 200 bp and above were considered.

## Data Availability

This Whole Genome Shotgun project has been deposited at DDBJ/ENA/GenBank under the accession JBLOOZ000000000. The version described in this paper is version JBLOOZ010000000. The sequence reads were submitted to the NCBI Sequence Read Archive (SRA) under the accession number SRX28200374.
